# PLA/PBSA Biocomposites Reinforced with Tangerine Tree-Derived Agro-Industrial Waste for Rigid Packaging: Effect of Extraction Treatment on Morphology and Thermo-Mechanical Performance

**DOI:** 10.3390/polym18121553

**Published:** 2026-06-22

**Authors:** Francesca Cartoni, Viola Berrugi, Aouatif Aboudia, Morad Chadni, Vito Gigante, Maria-Beatrice Coltelli

**Affiliations:** 1 Department of Civil and Industrial Engineering, University of Pisa, 56122 Pisa, Italy; francesca.cartoni@unipi.it (F.C.); violaberrugi@gmail.com (V.B.); vito.gigante@unipi.it (V.G.); 2 Laboratoire Bioresources et Securité Sanitaire des Aliments, Faculté des Sciences et Techniques, Université Cadi Ayyad, Marrakesh 40000, Morocco; a.aboudia@uca.ac.ma; 3 Université Paris-Saclay, AgroParisTech, URD Agro-Biotechnologies Industrielles, CEBB, 51110 Pomacle, France; morad.chadni@agroparistech.fr; 4 National Interuniversity Consortium of Materials Science and Technology (INSTM), Research Unit of University of Pisa, 56122 Pisa, Italy

**Keywords:** biocomposites, lignocellulosic fillers, tangerine tree, agro-industrial residues, extraction treatment

## Abstract

Bio-based and biodegradable polymer composites based on polylactic acid (PLA) and polybutylene succinate-co-adipate (PBSA) were developed for rigid food packaging applications. Agro-industrial residues consisting of ground leaves and branches derived from tangerine tree cultivation (pruning) were used as fillers at high loading (30 wt%) before (PRE) or after (POST) extraction of bioactive compounds. The influence of blend composition (PLA/PBSA 60/40 and 30/70), filler extraction, and the addition of antioxidants (0.5 wt%) on material properties was systematically investigated. Composites were processed via extrusion and injection molding and characterized through FTIR, SEM, tensile testing and thermal analysis. The results show that polymer blend morphology affects mechanical behavior, with co-continuous structures (60/40) exhibiting improved ductility compared to dispersed systems (30/70). The incorporation of lignocellulosic residues increased stiffness but reduced elongation at break. Extraction treatment significantly modified filler morphology and interfacial interactions, slightly improving dispersion and processability. The effect of the extracted bioactive compounds on the thermal stabilization of biocomposites was also investigated. Overall, the findings demonstrate the potential of combining biodegradable polymer blends with treated agricultural residues to produce sustainable rigid packaging materials while supporting a bio-circular approach. In fact, preliminary extraction of valuable compounds from tangerine pruning waste appears to be a convenient strategy for its efficient cascade valorization.

## 1. Introduction

The extensive use of fossil polymers in single-use packaging applications has led to severe environmental concerns due to their persistence, low recycling rates, and accumulation in natural ecosystems [1,2,3]. Packaging accounts for a significant fraction of global plastic production, with a large proportion designed for short-term use and rapid disposal [4,5]. The urgent need to reduce plastic waste and transition toward more sustainable materials has driven increasing research efforts in the development of biodegradable and bio-based polymers [6,7,8].

Among these, polylactic acid (PLA) is one of the most widely studied and commercially available biodegradable polymers, derived from renewable resources such as corn starch or sugarcane [9,10,11]. PLA exhibits good mechanical strength, transparency, and barrier properties, making it attractive for packaging applications [12,13,14,15]. However, its inherent brittleness, low impact resistance, and limited thermal stability restrict its use in rigid packaging systems such as trays [15,16,17,18,19].

To overcome these limitations, blending PLA with more ductile biodegradable polymers has been widely explored [19,20,21,22]. Polybutylene succinate-co-adipate (PBSA) is a flexible aliphatic polyester characterized by high elongation at break and good processability [23,24]. PLA/PBSA blends have shown improved toughness and flexibility compared to neat PLA, making them suitable candidates for packaging applications requiring enhanced mechanical performance [25,26,27]. Nevertheless, the immiscibility between PLA and PBSA often leads to phase-separated morphologies, which strongly influence the final properties of the blends [20,28], including recyclability [29] and compostability [30]. Considering these aspects, the PLA/PBSA 60/40 blend was found to be both recyclable and home-compostable [31,32]. Recently, the PLA/PBSA 60/40 blend was compared with the PLA/PBSA 30/70 blend. The latter exhibited comparable mechanical properties together with enhanced resistance to moisture transfer [33], highlighting its potential for packaging perishable and moisture-rich foods. These findings indicate that both formulations exhibit promising mechanical and barrier properties, making them suitable candidate matrices for composites containing high filler loadings. In this context, biocomposites based on PLA/PBSA blends containing wheat bran [34], rice bran [35], coffee silverskin [36], or microfibrillated cellulose [37] have already been investigated. In parallel, the incorporation of lignocellulosic fillers derived from agricultural and agro-industrial waste has gained increasing attention as a strategy to reduce material cost, improve stiffness, and enhance biodegradability [38,39,40,41,42]. Agricultural residues such as husks, fibers, and pruning waste are abundant, renewable, and often underutilized resources that can be valorized in polymer composites [43,44,45]. The addition of such fillers typically increases the elastic modulus but may reduce ductility due to poor interfacial adhesion and stress concentration effects [46,47,48,49].

In Mediterranean regions, large quantities of lignocellulosic residues are generated from fruit cultivation, particularly from pruning operations of citrus trees [50,51,52]. These residues are mainly composed of cellulose, hemicellulose, and lignin and can be processed into powders that are suitable for composite production [53,54,55]. Their use in biodegradable polymer matrices represents an effective approach to implement circular economy principles by transforming waste into value-added materials [56,57].

An emerging strategy in this field involves the extraction of bioactive compounds, such as antioxidants and phenolic substances, from agricultural residues prior to their use as fillers [58,59,60,61,62]. These compounds can be employed in active packaging applications to improve food preservation, while the remaining solid residues can still be used as reinforcing fillers [63,64,65]. However, the extraction process can significantly alter the chemical composition, morphology, and surface properties of the residues, thereby affecting their interaction with the polymer matrix [66,67,68].

In particular, the removal of low-molecular-weight compounds and extractives may lead to changes in filler surface roughness, porosity, and polarity, which can influence dispersion, interfacial adhesion, and overall composite performance [68,69,70]. Despite the growing interest in this dual valorization approach, the effect of extraction treatments on the structure–property relationships of polymer composites remains insufficiently explored [71,72].

In addition to mechanical performance, the rheological behavior of polymer composites is a critical parameter for industrial processing [73,74]. The incorporation of lignocellulosic fillers generally increases melt viscosity and reduces flowability, which can limit processability, especially in high-filler-content systems [75,76]. Conversely, changes in filler morphology induced by extraction processes may affect melt fluidity and facilitate processing [77,78]. Understanding the interplay between filler treatment, melt rheology, and final properties is therefore essential for the design of optimized materials.

Moreover, the use of antioxidants extracted from agricultural waste as potential stabilizers in biocomposites containing the corresponding extraction residues has not yet been explored [79]. Tangerine trees are widely cultivated in Mediterranean countries and other regions worldwide. Although the antioxidant properties of *Citrus reticulata* have been extensively investigated, most studies have focused on the fruits and fruit-processing by-products, such as peels, pomace, and seeds [80,81,82,83,84], whereas tree-derived residues (e.g., leaves and pruning biomass) remain comparatively underexplored [85,86,87]. Recently, Cravotto and co-workers [88] extracted bioactive compounds from tangerine tree leaves and branches, obtaining antioxidant-rich extracts mainly composed of hesperidin. The optimized process was based on subcritical water extraction.

In this context, the present work investigates PLA/PBSA-based biocomposites reinforced with tangerine-derived agro-industrial residues for rigid packaging applications, specifically trays. The study focuses on the effect of extraction treatment on filler characteristics and its influence on composite morphology, melt fluidity, and mechanical performance. Two blend compositions (PLA/PBSA 60/40 and 30/70) and a high filler loading (30 wt%) were considered to evaluate structure–property relationships under conditions that are relevant to industrial applications.

The novelty of this work lies in the comprehensive analysis of the morphology and rheological, mechanical, and thermal properties of biocomposites containing pre- and post-extraction tangerine tree residues. The influence of the extracted antioxidants on the thermal stability of the materials was also evaluated. The results provide new insights into the role of treated agricultural residues in biodegradable composites and contribute to the development of sustainable materials for rigid food packaging applications.

## 2. Materials and Methods

### 2.1. Materials

The PLA used in this study is Luminy LX175 (Gorinchem, The Netherlands), purchased from Total Corbion. It is an extrusion PLA grade that contains about 4% of D-lactic acid units [density: 1.24 g/cm^3^; melt flow index (MFI) (performed at 190 °C and 2.16 kg, [89]): 3 g/10 min].

The PBSA used in this work is BioPBS FD92PM, purchased from Mitsubishi Chemical Corporation (Tokyo, Japan) [density of 1.24 g/cm^3^; MFI (ISO 1133-A at 190 °C, 2.16 kg, [89]): 4 g/10 min].

The tangerine tree waste (TTW) of the Nadorcott variety comes from Morocco. In the form of a finely ground powder, it was obtained by shade-drying a mixture of leaves and stems obtained from clementine prunings collected on a farm near Marrakech.

The powder was extracted by critical water extraction to separate antioxidants (AOs) [85] so that an extracted fraction and an abundant POST-extraction residue were obtained. Tangerine tree waste obtained from pruning was used as fillers in both PRE- and POST-extraction forms.

### 2.2. Preparation of Composites

After drying all the materials at 60 °C in a ventilated oven (Binder, Tuttlingen, Germany) for 16 h, a laboratory-scale twin-screw mixer–extruder (Thermo Fischer Scientific HAAKE Mini-Lab II, Waltham, MA, USA) was used for preparing biocomposites. The system is based on a conical twin-screw compounder with an integrated recirculation channel, which, together with a bypass valve, allows for precise control of residence time. At the end of the test, the bypass valve can be opened, and the sample is extruded as a filament. The torque values of the different formulations were determined. Composites were prepared at 185 °C, with a screw speed of 110 rpm and a time of 1 min. Each miniextrusion trial was repeated several times until enough specimens and material had been produced for each subsequent test. Different formulations have been prepared (Table 1).

The HAAKE MiniJet II, which is a piston injection molding system designed for laboratory-scale sample preparation, was used in combination with the HAAKE MiniLab II microcompounder for the production of dog-bone specimens (Haake III type dog-bone tensile bars: width 10 mm, width in the narrow section 4.8 mm, thickness 1.35 mm, and length 90 mm) for the characterization of the composites. The set parameters, different for the composites having PLA/PBSA 60/40 or PLA/PBSA 30/70 matrix, are reported in Table 2.

**Figure 1:**
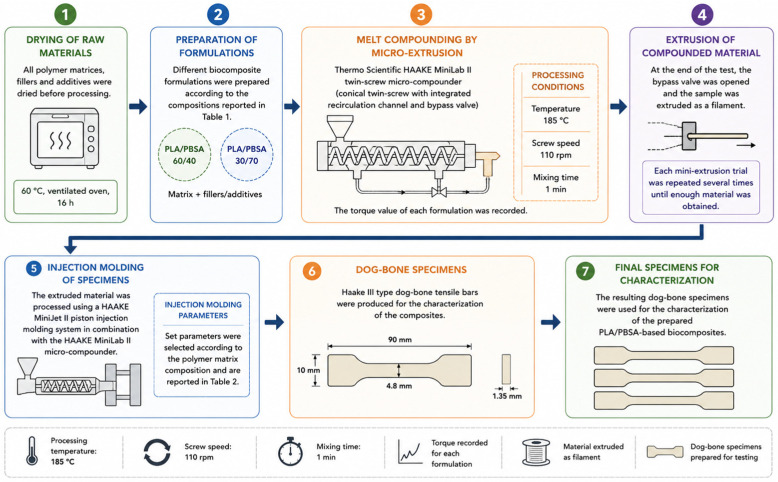
Scheme of the preparation of PLA/PBSA-based biocomposites containing tangerine waste PRE- or POST-extraction and extracted antioxidants in 7 phases.

A scheme for preparation of biocomposites is reported in Figure 1.

### 2.3. Fourier Transform Infrared Spectroscopy (FTIR)

FTIR analyses were performed using a Thermo Scientific Nicolet Apex spectrometer equipped with an ATR (attenuated total reflectance) Smart Itx accessory with a diamond crystal. Spectra were collected in the range 4000–550 cm^−1^. FTIR spectra of the dog-bone injection-molded specimens were analyzed in order to highlight the effects of the presence of the extracted and non-extracted residues and the antioxidants.

### 2.4. Differential Scanning Calorimetry (DSC)

Thermal properties were investigated using a DSC Q200 instrument (TA Instruments, New Castle, DE, USA). Approximately 10 mg of each sample was sealed in aluminum pans and subjected to the following thermal cycle: 1: jump to −70.00 °C; 2: isothermal step for 1.00 min; 3: ramp 10.00 °C/min to 200.00 °C; 4: isothermal step for 1.00 min; 5: ramp 20.00 °C/min to −70.00 °C; 6: isothermal step for 1.00 min; 7: ramp 10.00 °C/min to 200.00 °C; 8: isothermal step for 1.00 min. The heating/cooling rate was fixed at 10 °C/min under nitrogen atmosphere to prevent oxidation. PLA and PBSA crystallinity were calculated by using Equation (1).

(1) $$X_{cc,PLA\left(or\;PBSA\right)}=\frac{{∆H}_{m,PLa(or\;PBSA)}-{∆H}_{cc,PLA(\;or\;PBSA)}}{{∆H}_{m,PLA\left(or\;PBSA\right)}^{0}\cdot wt.\%PLA\;(or\;PBSA)}$$

### 2.5. TGA

Thermogravimetric analysis was performed with a PerkinElmer Pyris TGA 4000 thermogravimetric analyzer (PerkinElmer, Waltham, MA, USA) at a heating rate of 10 °C/min under nitrogen purge (30 mL/min) from room temperature to 815 °C, then with air as purge gas up to 850 °C. About 10 mg of each sample was used for the analysis. The software Pyris TGA 9 was used for thermogram elaborations.

### 2.6. SEM

The injection-molded specimens were cryo-fractured in liquid nitrogen, and the fractured surfaces were coated with platinum via sputtering. A Scanning Electron Microscopy (SEM) analysis was then carried out using an FEI Quanta 450 FEG instrument (Graz, Austria). The dimensional analysis was carried out by using the Image J 1.54g software on 3 micrographs having a magnification of 800×, determining the maximum length of 120 filler fibers.

### 2.7. Tensile Tests

The tests were carried out on injection-molded dog-bone specimens [90] using the universal testing machine MTS Criterion model 43 (Eden Prairie, MN, USA). The set width was 5 mm, the thickness 1.6 mm, the grip separation 25 mm, the test rate 10 mm/min and the data acquisition rate 10.0 Hz. At least 6 specimens were tested. Given the high variability of the material, the width and thickness of each specimen were measured prior to each test using a caliper. With the present test, stress at break and deformation at break were evaluated.

### 2.8. Dynamic Mechanical Analysis (DMA)

The dynamic mechanical analysis (DMA) was performed on a Gabo Eplexor^®^ 100 N (Gabo Qualimeter GmbH, Ahlden, Germany). DMA analysis was carried out to evaluate the elastic modulus as the storage modulus (E’) determined in tensile configuration at room temperature of the dog-bone specimens. The assumption E ≈ E’ is robustly supported because the viscous component (loss modulus) at room temperature is sufficiently small. Consequently, the difference between the complex modulus and the storage modulus becomes negligible, making E’ a reliable approximation of the elastic modulus [91,92]. In this case, the modulus was calculated by performing three tests for each composition. The reference length was 20 mm, and the specimens used were 40 mm long. The width and thickness of each specimen were measured for each formulation with a caliper.

## 3. Results and Discussion

### 3.1. Composition and Thermal Stability of Tangerine Tree Waste and Its Extraction Products

The subcritical water extraction of ground tangerine leaves and stems obtained from pruning produced a mixture of compounds, as revealed by LC–MS/MS analysis. These included hesperidin, naringin, poncirin, and polymethoxylated flavones such as nobiletin and tangeretin, with hesperidin identified as the most abundant component [88]. The FTIR spectrum of the extract shows characteristic bands consistent with pure hesperidin. However, slight shifts in band positions are observed, notably in the –OH stretching and C–H stretching regions. A key difference is the appearance of a strong peak at 1050 cm^−1^ in the extract, attributed to C–O and C–C vibrations associated with pyranose rings or flavonoid structures, indicating the presence of sugars or polyphenolic compounds. A comparison of the spectra from the raw material and extraction residue highlights significant changes in the region that are typical of polyphenols in lignocellulosic matrices, suggesting that the extraction has significantly reduced the polyphenol content. The increased intensity of the –OH band in the POST-extraction residue suggests that extraction under high temperature and pressure induces hydrolysis and partial depolymerization, generating additional hydroxyl groups [88].

**Figure 2:**
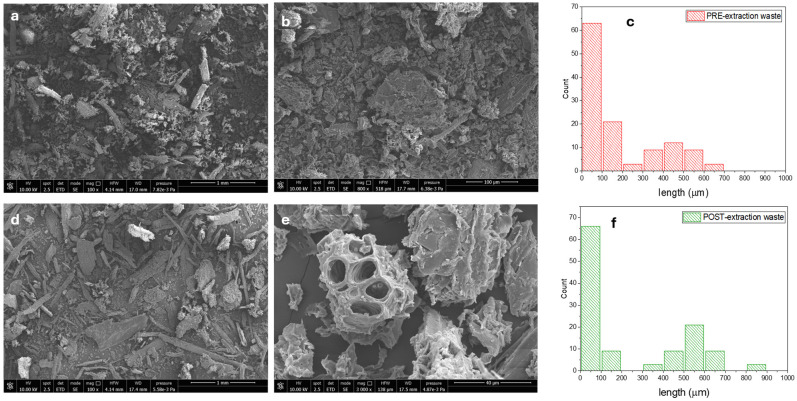
SEM micrographs of tangerine tree pruning powder: (**a**) PRE-extraction residue at 100× magnification; (**b**) PRE-extraction residue at 800× magnification; (**c**) distribution of particle length of PRE-extraction residue; (**d**) POST-extraction residue at 100× magnification; (**e**) POST-extraction residue at 1000× magnification; (**f**) distribution of particle length of post-extraction residue.

The dimensional analysis of the main fiber length yielded values of 177 ± 196 µm for the pre-extraction residue and 232 ± 260 µm for the post-extraction residue, indicating that the broad size distribution was not significantly affected by the extraction process. In contrast, the morphology of the residue was markedly influenced by extraction as cavities were observed in the post-extraction material that were absent in the pre-extraction sample (Figure 2). Thus, extraction induced changes both in the surface composition and morphology of the residue by removing specific compounds from its surface.

**Figure 3:**
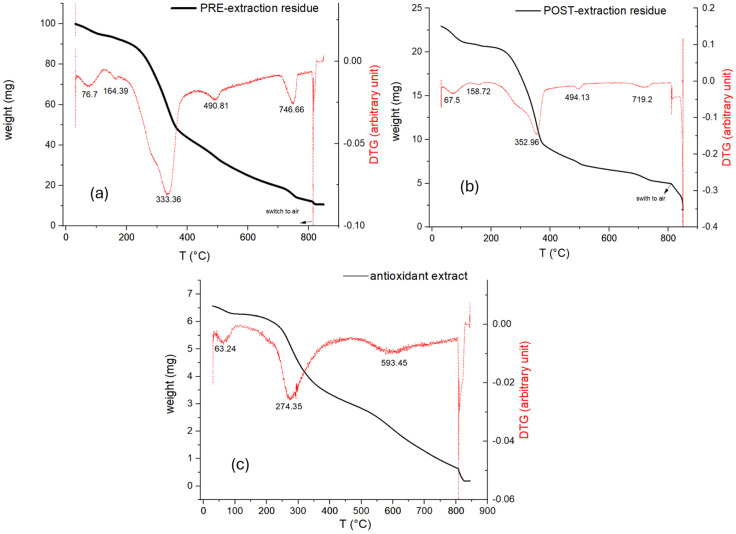
TGA thermograms including derivative DTG curved (in red) recorded for tangerine tree pruning waste: (**a**) PRE-extraction residue; (**b**) POST-extraction residue; (**c**) antioxidant extract.

Regarding thermal stability, the PRE-extraction tangerine tree residue as well as the extracted and POST-extraction fraction were analyzed by thermogravimetry. Both the PRE-extraction and POST-extraction residues showed five mass loss steps, and the weight percentages of mass loss as well as residue percentages are reported in Table 3 and Figure 3.

The first one (Step 1), occurring at 65–80 °C, may be attributed to the release of humidity. The second one (Step 2), occurring at about 160 °C, may be attributed to the beginning of pyrolysis, mainly pertaining to more volatile components [93] or degradation of hemicellulose [94]. The main degradation step is the third one (Step 3), showing a maximum velocity at 333 °C for the PRE-extraction and 353 °C for the POST-extraction residues, which can be attributed to hemicellulose [95] or hemicellulose and cellulose [88,96,97]. Galiwango et al. (2019) [98] reported that cellulose thermal decomposition spans approximately in the 150–500 °C range, with a strong dependence on biomass composition. For example, decomposition occurs at 250–400 °C for *Mentha arvensis*, 300–360 °C for maize straw, and 230–315 °C for cotton. The step occurring to about 490 °C (Step 4) is attributable to the decomposition of the lignin fraction [99,100]. As suggested by Butnaru et al. [100], this stage involved the advanced charring reactions of the thermally degraded lignin, with the release of hydrogen, methane and carbon monoxide/carbon dioxide. Interestingly the mass loss related to this step is lower in the POST-extraction residue. This can be attributed to the action of the extracting subcritical water, which may induce partial decomposition of lignin [101,102,103], inducing it to degrade at lower temperature, reasonably with cellulose.

The mass loss at about 700 °C (Step 5) may correspond to the thermal decomposition of the inorganic compounds, such as carbonates, which are usually present in large quantities in biomass samples [104]. The product of this step is a carbonaceous residue of pyrolysis, and, for the POST-extraction sample, this residue is more abundant, suggesting that the removal of compounds at lower molecular weight and the modification due to the attack of subcritical water may affect the pyrolysis path significantly.

The further mass loss represented the burning of the carbon when the flow gas was shifted at 815 °C from inert nitrogen to oxidative air. The final residue represents the remaining inorganic ash, and it is slightly lower for the POST-extraction residue.

The thermogravimetric trend of the AO extract is significantly different and less complex than the ones of residues. The extracted fraction, consisting mainly of hesperidin, shows a temperature corresponding to the highest degradation velocity (274 °C), similar to that of hesperidin [105]. Then only another mass loss step can be revealed, centered at 593 °C, attributable to cracking and condensation involving the aromatic structures typical of the extract, thus producing aromatic hydrocarbons, as suggested by the significant residue of 10.10%. After the step in air, the extract showed the lowest inorganic residue, in agreement with the results about PRE-extraction and POST-extraction residues.

### 3.2. Composition and Morphology of Biocomposites

**Figure 4:**
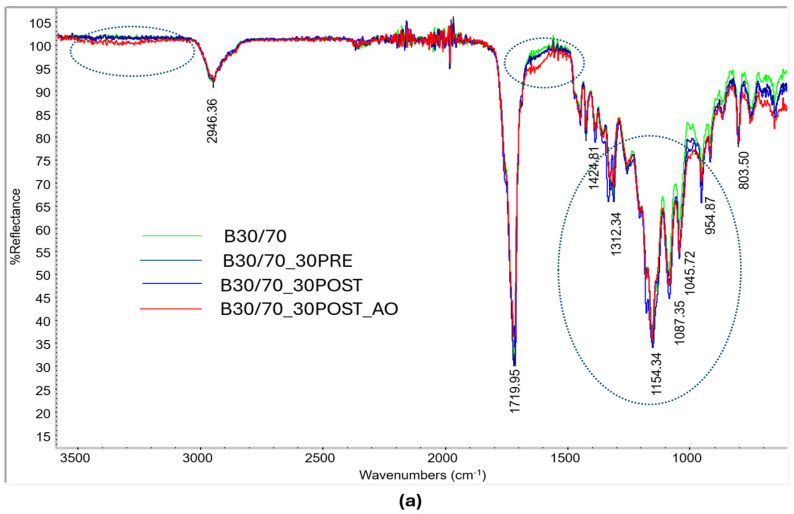

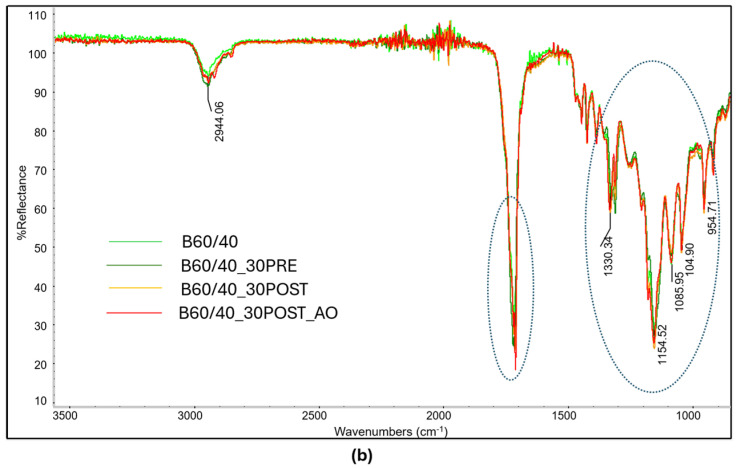
Infrared ATR spectra of the biocomposites containing tangerine tree-derived powder and antioxidants: (**a**) biocomposites based on PLA/PBSA 30/70; (**b**) biocomposites based on PLA/PBSA 60/40.

The ATR–FTIR spectra collected from the injection-molded specimens display the characteristic absorption bands of PLA/PBSA blends and their corresponding biocomposites (Figure 4). The bands in the 2995–2940 cm^−1^ region are attributed to C–H and CH_3_ stretching vibrations of PLA and CH_2_ stretching of PBSA. The strong absorptions observed between 1750 and 1710 cm^−1^ correspond to the carbonyl (C=O) stretching of the two polyesters, with PLA exhibiting a peak at ~1748 cm^−1^ and PBSA at ~1725 cm^−1^ [106]. The region between 1455 and 1370 cm^−1^ is associated with CH_3_ and CH_2_ bending vibrations, while the 1270–1080 cm^−1^ region is characteristic of C–O–C stretching of ester groups.

In the spectra of the biocomposites, additional weak bands appear in the 1600–1500 cm^−1^ region, which can be attributed to C=C vibrations related to lignin structures and phenolic compounds present in the plant-derived residues. A broad low-intensity band in the 3600–3200 cm^−1^ range is assigned to O–H stretching vibrations, arising from hydroxyl groups in cellulose, hemicellulose, lignin, and absorbed moisture; this feature is particularly evident in the residue-containing samples.

Variations in the fingerprint region are observed, especially in samples containing antioxidants (PRE and POST_AO), suggesting that the residues and associated bioactive compounds influence the chemical structure and interactions within the composite.

For the PLA/PBSA 60/40 blend, compared to the 30/70 composition, a greater contribution from PLA is evident, as indicated by the increased intensity of the C=O band at ~1748 cm^−1^ and the more defined peaks in the fingerprint region (notably at ~870 and ~760 cm^−1^). The presence of PBSA is confirmed by the broadening of the carbonyl band toward ~1720 cm^−1^, as well as by C–H stretching bands at ~2950 and ~2850 cm^−1^ and characteristic absorptions around ~600 and ~660 cm^−1^.

Upon incorporation of the residues, subtle changes are observed in the 3500–3200 cm^−1^ region, consistent with the introduction of hydroxyl-containing plant constituents such as alcohols and phenols. This effect is less pronounced in composites containing POST-extraction residues, indicating the partial removal of phenolic compounds during extraction. The addition of pure antioxidants at low concentration (0.5 wt%) is not clearly detectable in the spectra. As also observed for the 30/70 blend, the presence of antioxidants leads to slight modifications in the 1250–800 cm^−1^ region, which may suggest interactions with the polymer matrix.

Overall, the FTIR analysis confirms the coexistence of polymeric components and lignocellulosic residues while also indicating that the extraction process partially removes phenolic and other bioactive compounds from the tangerine-derived material.

**Figure 5:**
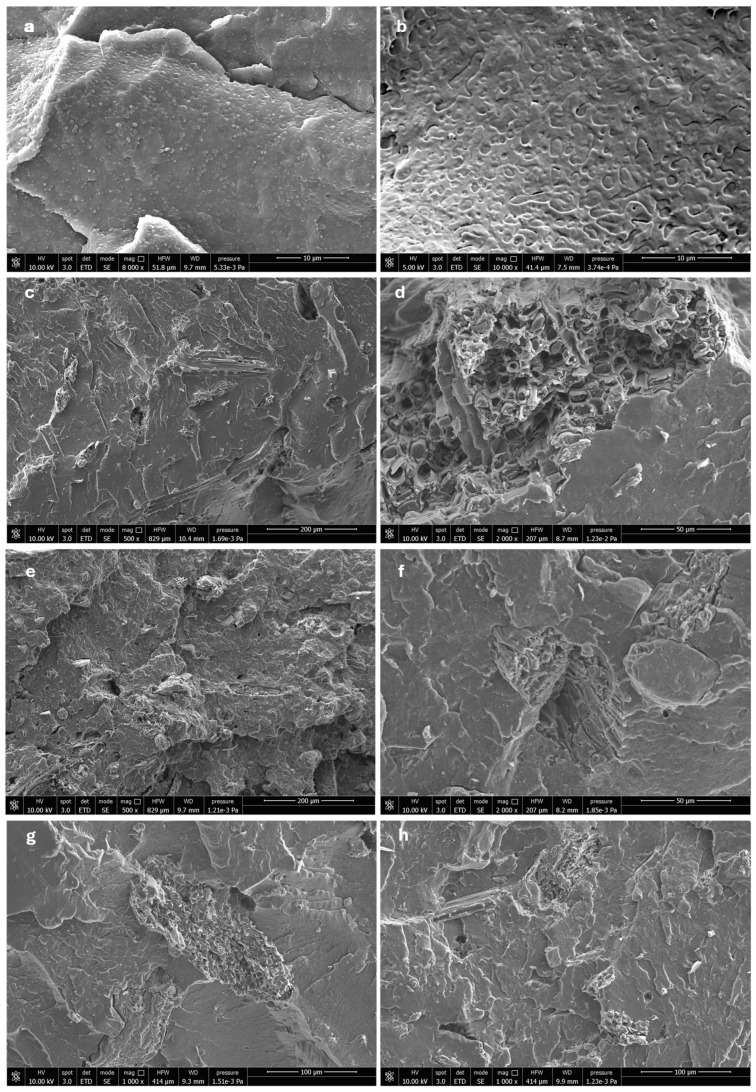
SEM micrographs of biocomposites: (**a**) B30/70; (**b**) B60/40; (**c**) B30/70_30PRE; (**d**) B30/70_30POST; (**e**) B60/40_30PRE; (**f**) B60/40_30POST; (**g**) B30/70_30PRE_AO; (**h**) B60/40_30PRE_AO (Table 1).

The SEM micrographs of the produced biocomposites reveal the phase morphology of the polymer blends as well as the dispersion of tangerine tree-derived fillers within the matrices. The 30/70 PLA/PBSA blend exhibits a dispersed morphology (Figure 4a), characterized by small PLA droplets distributed within the continuous PBSA phase. In contrast, the 60/40 blend displays a co-continuous structure (Figure 5b), where PLA forms elongated interconnected domains rather than isolated inclusions, indicating partial phase continuity. This morphology, consistent with previous studies [20,31], may promote improved interfacial adhesion and consequently influence the mechanical performance compared to systems with discrete PLA domains. Figure 5c,d compare the incorporation of PRE- and POST-extraction residues within the 30/70 matrix. The PRE-extraction sample shows good interpenetration between the fibers and the polymer matrix, not evidencing significant detachment between them. In the POST-extraction sample, the presence of cavities within the residues—attributed to the extraction process—is evident; nevertheless, adhesion to the polymer matrix remains effective. Similarly, in the 60/40 matrix (Figure 5e,f), the fibers appear well integrated, confirming that good filler–matrix adhesion is maintained regardless of blend composition. Evidence of polymer infiltration into the fibrous structure is also observed, which may affect the overall composite properties. The presence of ellipsoidal and interconnected PBSA domains further supports the co-continuous morphology of the 60/40 blend. No preferential localization of either polymer phase at the filler surface is detected. The antioxidant additive (0.5 wt%) is not visibly distinguishable in the micrographs. However, the images of the biocomposites containing the additive (Figure 5g,h) further confirm both the effective filler–matrix adhesion and the distinct morphological characteristics of the 30/70 and 60/40 blends.

### 3.3. Processing Aspects and Mechanical Characterization

**Figure 6:**
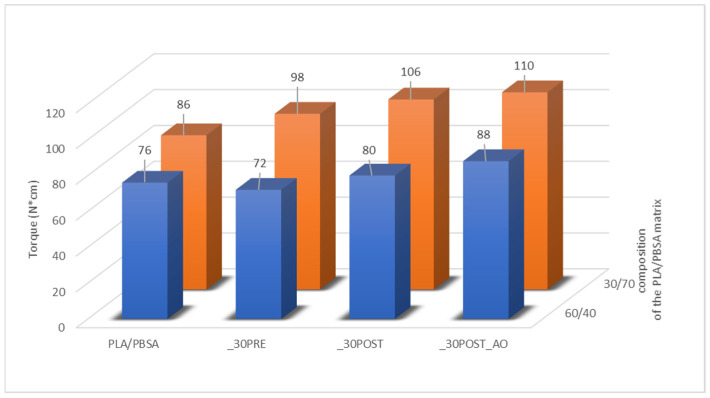
Torque values recorded during extrusion of biocomposites containing tangerine pruning waste residues (PRE- and POST-extraction) and their extracts. Samples based on PLA/PBSA 30/70 blends are shown in orange, whereas samples based on PLA/PBSA 60/40 blends are shown in blue.

The torque values recorded during extrusion provide an indirect indication of melt viscosity (Table 1). A clear difference is observed between the two blends: the PLA/PBSA 60/40 composition, richer in PLA, exhibits lower torque values compared to the 30/70 blend, which contains a higher proportion of PBSA (Figure 6). This suggests that the 30/70 system is significantly more viscous under the applied processing conditions. Such behavior is consistent with reports in the literature [32,107], where PBSA has been shown to undergo chain extension or branching reactions at elevated temperatures, leading to increased melt viscosity.

For the composite formulations, the incorporation of residues generally results in an increase in torque, indicating higher viscosity. In the PLA/PBSA 30/70 system, a progressive increase in viscosity is observed when moving from PRE to POST and POST_AO biocomposites. Conversely, in the 60/40 matrix, the addition of PRE-extraction residue leads to a slight decrease in torque. Notably, for both matrices, the inclusion of antioxidants results in a significant increase in torque.

This behavior suggests that, under extrusion conditions, antioxidants may contribute to the stabilization of polymer chains and potentially promote chain extension or branching reactions, thereby increasing molecular weight [108]. In extruding biopolyesters and their biocomposites, a decrease in torque is typically associated with polymer degradation due to chain scission, whereas an increase indicates the occurrence of coupling reactions. Overall, the results indicate that the biocomposites exhibit higher viscosity than the neat matrices, and that the presence of antioxidants contributes to a more stable and viscous melt, suggesting a potential stabilizing action of this extract.

**Figure 7:**
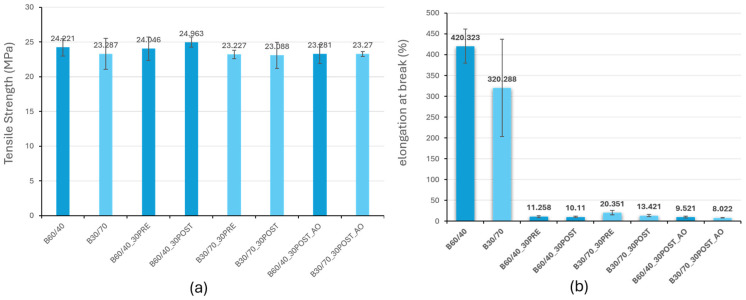
Tensile characterization of samples: (**a**) Tensile strength and (**b**) elongation at break of biocomposites: dark blue bars indicate those based on PLA/PBSA (60/40), and light blue bars indicate those based on PLA/PBSA (30/70).

Tensile testing (Figure 7a,b) revealed that the neat polymer matrices exhibit very high elongation at break, exceeding 400%. Although PBSA is inherently more ductile, the PLA/PBSA (30/70) matrix shows lower elongation at break compared to the PLA/PBSA (60/40) system. This behavior can be attributed to differences in phase morphology, as evidenced by the SEM analysis (Figure 4). In the 30/70 composition, the lower PLA content (30%) likely promotes a morphology in which PLA-dispersed domains may act as stress concentrators, increasing susceptibility to crack initiation and resulting in reduced ductility. In contrast, the 60/40 blend exhibits a more co-continuous structure, where both polymer phases are interconnected and contribute to load transfer, thereby enhancing deformability.

The tensile strength values (Figure 7a) are comparable across all the formulations, ranging from approximately 23 to 25 MPa. Variations in matrix composition do not lead to significant differences in tensile strength or stiffness, indicating that these properties remain largely unaffected. However, the 60/40 formulations consistently show slightly higher yield and tensile strength than the 30/70 systems, suggesting that increased PLA content contributes modestly to strength enhancement.

With regard to the composites, the incorporation of tangerine tree residues (PRE, POST, and ANTIOX) results in a marked reduction in ductility, with elongation at break decreasing to approximately 8–20% (Figure 7b). This behavior indicates that the fillers act as embrittling agents, limiting the material’s capacity for plastic deformation. Agglomeration occurring at the adopted high filler loading may contribute to this embrittlement. The extraction process does not appear to significantly influence the mechanical properties as no substantial differences are observed among the PRE, POST, and antioxidant-treated samples.

The addition of residues and antioxidants induces only minor variations in tensile strength, suggesting that these additives do not significantly compromise the structural integrity of the material within the elastic regime. Despite the pronounced reduction in ductility caused by residues (PRE/POST) and antioxidants (ANTIOX), the ultimate tensile strength remains largely unchanged across all the formulations. Notably, those formulations containing the antioxidant exhibit the greatest degree of embrittlement, reflected in the lowest elongation at break and slightly reduced tensile strength. This observation may indicate suboptimal dispersion of the antioxidant within the polymer matrix.

The elastic modulus of the biocomposites was determined by dynamic mechanical analysis (DMA), taken as the storage modulus (E’) evaluated at room temperature, as explained in the Materials and Methods Section. The two neat matrices, PLA/PBSA (30/70) and PLA/PBSA (60/40), exhibit comparable stiffness, which can again be attributed to their morphological characteristics. The incorporation of additives (PRE, POST, and ANTIOX) in both compositions leads to a significant increase in stiffness, as evidenced by higher elastic modulus values. In particular, for the 30/70 matrix, the addition of POST residues results in the highest stiffness, whereas, in the 60/40 system, the PRE residues yield the stiffest formulation.

**Figure 8:**
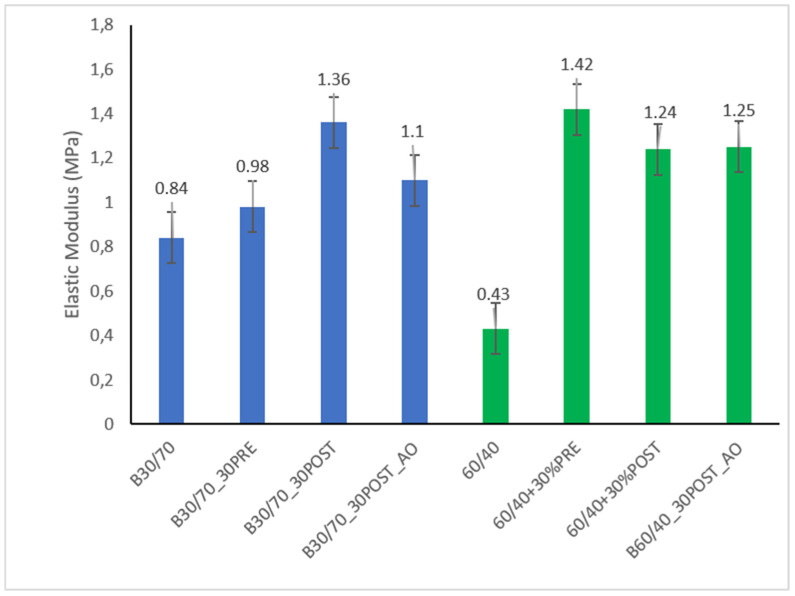
Elastic moduli, measured by DMA analysis, of the different biocomposites. Samples based on PLA/PBSA 30/70 blends are shown in blue, whereas samples based on PLA/PBSA 60/40 blends are shown in green.

While tensile testing showed that yield strength and ultimate tensile strength remain nearly unchanged, the DMA results indicate a substantial increase in elastic modulus (Figure 8). This suggests that the materials become significantly stiffer without a corresponding improvement in strength at break. The additives therefore appear to exert a dual effect, increasing stiffness while simultaneously promoting embrittlement, thereby reducing the material’s ability to undergo plastic deformation.

In summary, plant-derived residues and antioxidants act as reinforcing or stiffening agents in PLA/PBSA blends.

### 3.4. Thermal Analysis

**Figure 9:**
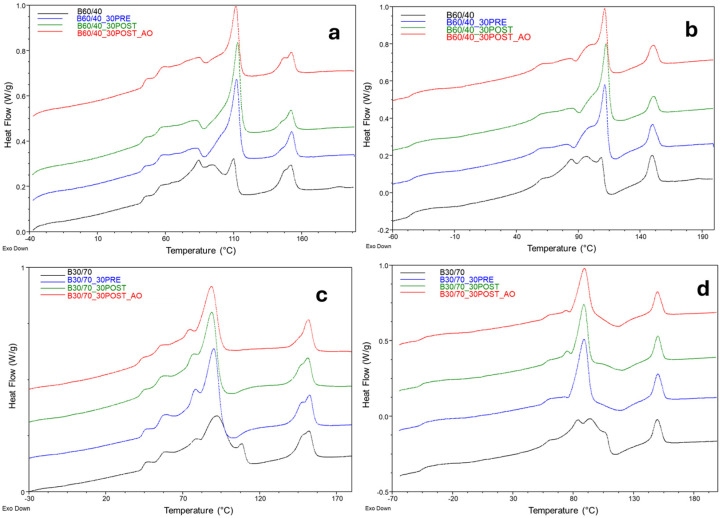
DSC results of biocomposites: (**a**) first heating scan for biocomposites based on blend PLA/PBSA 60/40; (**b**) second heating scan for biocomposites based on blend PLA/PBSA 60/40; (**c**) first heating scan for biocomposites based on blend PLA/PBSA 30/70; (**d**) second heating scan for biocomposites based on blend PLA/PBSA 30/70.

Both the DSC and TGA investigations were carried out on injection-molded specimens of the produced biocomposites. The DSC analysis indicates how the different additives could change the processing and the intrinsic properties of the materials (Figure 9).

The analysis of first heating thermograms can be useful to study the thermal phenomena that occurred during injection molding of specimens. Hence, this study can be significant for the potential further processing by injection molding of composites. The first heating thermograms of the PLA/PBSA (60/40) biocomposites (Figure 9a) show well-defined endothermic peaks corresponding to the melting temperatures of both PLA and PBSA. The glass transition of PLA appears to be at around 55 °C. The addition of residues does not significantly affect the mobility of the amorphous PLA phase as the glass transition temperature remains essentially unchanged.

Multiple melting peaks for PBSA were present in the region 60–120 °C, suggesting that, during rapid cooling in the mold, this polymer forms irregular crystals that melt at lower temperatures. In the composites the main melting peak is the one at the highest temperature (about 110 °C). This suggests that the filler can facilitate the formation of more regular crystals with respect to the B60/40 blend. Cold crystallization of PLA may still occur, but it is weak and difficult to quantify; therefore, the degree of crystallinity of the polymers could not be reliably determined due to the overlapping of different phenomena. Overall, no significant differences are observed among the various biocomposite formulations during the first heating scan.

The second heating thermograms, which reflect the intrinsic thermal properties after erasure of thermal history (Table 4), show a well-defined glass transition for PLA, unaffected by the presence of residues. This confirms that the multiple transitions observed during the first heating are likely related to processing conditions, particularly cooling during injection molding. The melting peaks of both polymers are clearly visible. The PBSA melting peak partially overlaps with the cold crystallization of PLA, which is especially evident in the neat matrix, where multiple exothermic peaks suggest crystallization occurring in distinct domains. The addition of residues reduced the extent of cold crystallization, with PBSA melting becoming the dominant feature. These results confirm that the residues exert a nucleating effect, promoting crystallization during cooling, although no significant differences are observed among the different residue types.

For the PLA/PBSA (30/70) biocomposites (Figure 9c), the first heating thermograms similarly show complex thermal behavior in the low-temperature region due to overlapping phenomena. The PLA glass transition appears as multiple steps in the range of 48–55 °C and remains largely unaffected by the addition of residues. Weak exothermic signals attributable to cold crystallization are observed, although their quantification is hindered by overlap with PBSA melting.

The melting peaks of both polymers are clearly identifiable. In the neat matrix, PBSA exhibits a double melting peak, with one peak around 108 °C (similar to the 60/40 blend) and a second around 90 °C, suggesting the presence of distinct crystalline populations. Upon addition of residues, the higher-temperature peak disappears. This behavior can be explained by both thermodynamic and morphological considerations. In the 30/70 blend, PBSA constitutes the continuous phase, resulting in a larger interfacial area between the PBSA crystals and the amorphous PLA phase. This can lead to the formation of less stable crystals with lower melting temperatures. In contrast, the co-continuous morphology of the 60/40 blend promotes the formation of more stable and well-developed crystals. The addition of plant residues appears to exert a nucleating effect on PBSA, leading to a more uniform crystalline structure and masking the double melting behavior. No significant differences are observed among the different additives.

The second heating thermograms of the PLA/PBSA (30/70) composites (Table 4) confirm these observations. After removal of thermal history, multiple peaks for PBSA melting disappeared, with no significant changes due to additives. The lower melting temperature of PBSA compared to the 60/40 system is maintained, consistent with the blend morphology. In the biocomposites, the melting peaks of both PLA and PBSA are well resolved, and the addition of residues stabilizes the PBSA melting behavior, which is more complex in the neat matrix. The ΔH_m_ of PLA in the PLA/PBSA 30/70-based composites increased significantly, even considering that the blend represented only 70% of the composite. This suggests that the filler facilitates the crystallization of PLA during the cooling or heating step, although PLA is dispersed in domains in these materials.

Exothermic peaks associated with the cold crystallization of PLA are observed. The presence of residues shifts the cold crystallization temperature to higher values, suggesting that they do not act as effective nucleating agents for PLA but rather restrict chain mobility, requiring higher thermal energy for crystallization. In contrast, the addition of antioxidant alone slightly lowers the cold crystallization temperature, indicating a modest nucleating effect compared to plant residues.

**Figure 10:**
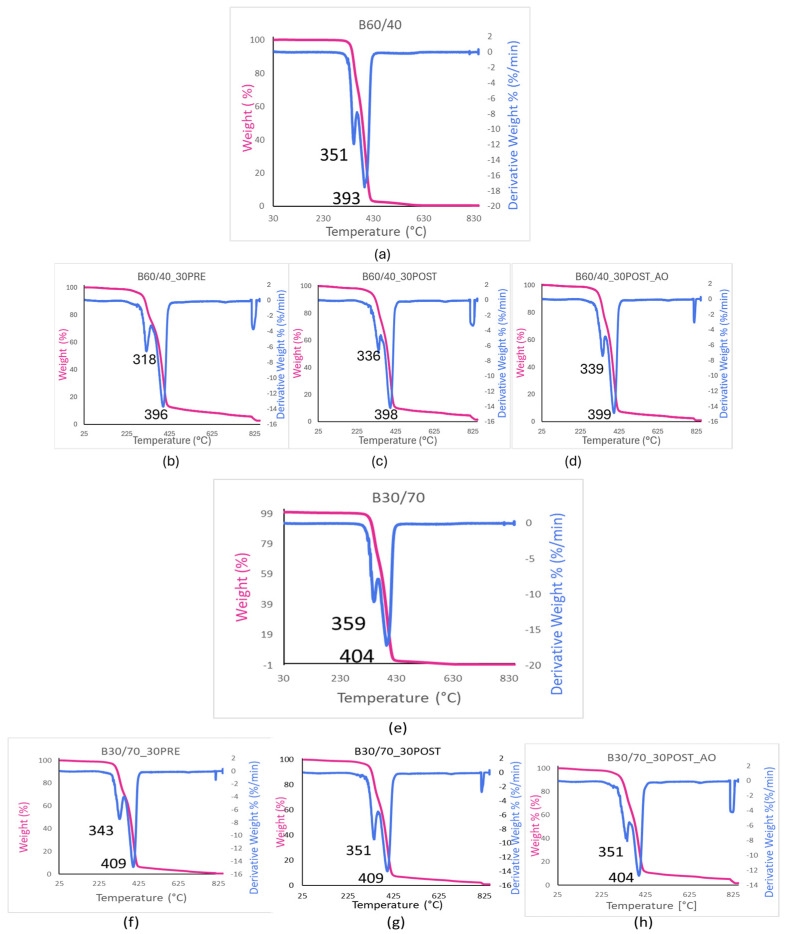
TGA thermograms and derivative curves (in red) of biocomposites, including derivative curves and main inflection point temperatures: (**a**) PLA/PBSA; 60/40 (**b**) B60/40_30PRE; (**c**) B60/40_30POST; (**d**) B60/40_30POST_AO; (**e**) PLA/PBSA 30/70 blend; (**f**) B30/70_PRE; (**g**) B30/70_POST; (**h**) B30/70_POST_AO.

A thermogravimetric analysis (TGA) was performed to evaluate the thermal stability of the biocomposites, considering the thermal behavior of the different additives (Section 3.1). The DTG curves were analyzed to identify the degradation steps (Figure 10), quantify the associated mass losses, and determine the residual mass under nitrogen and after switching to air (Table 5). Both the B60/40 and B30/70 matrices exhibit two main degradation steps, attributed to PLA [109,110,111] (351 °C and 359 °C for B60/40 and B30/70, respectively) and PBSA [112,113,114] (393 °C and 404 °C for B60/40 and B30/70, respectively).

The incorporation of 30 weight percentage tangerine tree waste (PRE) results in a clear reduction in thermal stability compared to the corresponding neat matrices. Specifically, the onset degradation temperature decreases by approximately 38 °C for B60/40_30PRE and by 30.5 °C for B30/70_30PRE. In addition, the peak temperature of the first degradation step (Figure 10b,f), associated with PLA degradation, is significantly shifted to lower values due to the presence of the PRE residue.

A notable result is that the use of the POST-extraction filler leads to improved thermal stability compared to PRE residues. The reduction in onset temperature is limited to 28 °C for B60/40 and 13.5 °C for B30/70. This indicates that residues subjected to subcritical water extraction introduce a lower degree of thermal instability. From an application perspective, this finding supports a valorization strategy in which citrus pruning waste is first used for the extraction of valuable antioxidants and the resulting POST-extraction residue is subsequently employed as a filler in biocomposites.

The addition of antioxidants derived from tangerine tree residues further influences thermal stability. In B60/40-based composites, the presence of antioxidants reduces the onset temperature by approximately 21 °C, indicating a stabilizing effect compared to PRE-filled and POST-filled systems. This effect is not observed in B30/70-based biocomposites. This behavior suggests that the antioxidant is more effective in stabilizing PLA, which is present in a higher proportion in the 60/40 blend, than PBSA. Consistently, PLA is known to undergo thermal degradation at lower temperatures than PBSA [20,115].

These results highlight the potential of antioxidants derived from citrus pruning not only for applications in the cosmetic, food, and personal care sectors but also as bio-based stabilizing additives for polymeric materials, offering a sustainable alternative to conventional fossil-based additives. The possibility that the antioxidants can protect the content of packaging from oxidation is also interesting [79,116,117].

It was observed that the residue obtained after degradation under nitrogen, as well as that measured after switching to air, had significantly lower values than the corresponding values predicted based on the degradation behavior of the filler (Table 2, Section 3.1). Specifically, the expected residues for the PRE-extraction powder were 3.67% (nitrogen) and 3.23% (air), while, for the POST-extraction powder, they were 6.59% (nitrogen) and 2.58% (air). This discrepancy suggests that thermal degradation at high temperature follows a different pathway when tangerine tree waste is embedded within a polymeric matrix. Notably, the reduction in residue percentage was more pronounced in biocomposites based on PLA/PBSA (30/70), indicating that the higher PBSA content may enhance the decomposition of the tangerine tree agrowaste investigated in this study.

## 4. Conclusions

This study investigated the production of biocomposites via miniextrusion using PLA/PBSA (60/40 and 30/70) matrices reinforced with residues from tangerine tree pruning, both before and after antioxidant extraction, and evaluated their rheological, thermo-mechanical, and thermal properties. Additionally, biocomposites containing 0.5 weight percentage of extracted antioxidant compounds were prepared and analyzed.

The mechanical characterization of dog-bone specimens indicated that the best performance was achieved by biocomposites based on the PLA/PBSA 60/40 matrix. As expected, the incorporation of lignocellulosic residues increased stiffness while reducing ductility, leading to higher modulus values and lower elongation at break. Furthermore, the presence of the filler increased melt viscosity during processing.

The addition of extracted antioxidants produced subtle effects in biocomposites. In particular, they appeared to mitigate the rate and intensity of thermal degradation, enhance melt viscosity and stability during extrusion, and exert a mild nucleating effect—especially on PBSA—resulting in a more uniform crystalline morphology.

Biocomposites containing post-extraction (POST) fillers showed slightly improved mechanical properties and delayed onset of thermal degradation compared to those with untreated residues. Overall, the results demonstrate that both the extracted antioxidants and the residual biomass can be effectively valorized in the development of biocomposites.

In conclusion, this work supports the development of sustainable biocomposite materials for rigid bio-based circular packaging applications, promoting the valorization of agricultural waste, which is particularly abundant in the Mediterranean region.

## Figures and Tables

**Table 1:** Composition of the composites expressed as percentage by weight.

Title 1	PLA	PBSA	TTW-PRE	TTW-POST	AO
B60/40	60	40	-	-	-
B30/70	30	70	-	-	-
B60/40_30PRE	42	28	30	-	-
B60/40_30POST	42	28	-	30	-
B30/70_30PRE	21	49	30	-	-
B30/70_30POST	21	49	-	30	-
B60/40_30POST_AO	41.7	28.3	30	-	0.5
B30/70_30POST_AO	20.85	28.3	-	30	0.5

**Table 2:** Processing parameters for the injection molding of biocomposites.

Title 1	PLA/PBSA 60/40 Matrix	PLA/PBSA 30/70 Matrix
Temperature	185 °C	185 °C
Injection Pressure	300 bar (7 s)	250 bar (7 s)
Counter Pressure	200 bar (3 s)	150 bar (3 s)
Mold Temperature	60 °C	50 °C

**Table 3:** Thermogravimetric analysis (TGA) weight loss of tangerine tree pruning powder and its derived fractions after extraction.

Sample	Step 1 (wt.%)	Step 2 (wt.%)	Step 3 (wt.%)	Step 4 (wt.%)	Step 5 (wt.%)	Residue N_2_ (wt.%)	Residue Air (wt.%)
PRE-extraction residue	5.62	2.58	49.13	22.85	7.58	12.23	10.76
POST-extraction residue	8.71	1.46	52.99	6.35	8.51	21.99	8.61
AO extract	4.54	-	48.08 ^1^	37.32 ^2^	-	10.1	3.25

^1^ This mass loss occurred at 274 °C (Figure 2c); ^2^ this mass loss occurred at 593 °C (Figure 2c).

**Table 4:** DSC data related to biocomposites.

	I Heating			II Heating				
Samples	T_m_, PLA (°C)	T_m_, PBSA (°C)	ΔH_m_, PLA ^1^ (J/g)	T_g_, PLA (°C)	T_m_, PBSA (°C)	T_cc_, PLA (°C)	T_m_, PLA (°C)	ΔH_m_, PLA ^1^ (J/g)
B60/40	125.25	110.42	8.13	56.28	108.52	-	149.55	12.98
B60/40_30PRE	152.83	112.24 ^1^	5.92	56.14	111.43	-	149.86	6.78
B60/40_30POST	152.35	113.16	4.79	56.19	112.44	-	150.95	4.19
B60/40_30POST_AO	152.75	111.84	5.55	56.22	111.33	-	150.18	7.30
B30/70	152.10	9.9/108.9	8.44	57.12	-	112.79	149.70	8.93
B30/70_30PRE	152.52	90.49	8.60	56.64	89.27	120.71	150.21	10.21
B30/70_30POST	151.48	89.12	7.79	57.04	89.02	120.34	150.04	8.30
B30/70_30POST_AO	151.74	89.01	6.47	56.69	89.66	116.65	149.82	7.99

^1^ The reported ΔH_m_ values are expressed as joules per gram of sample.

**Table 5:** TGA data of the biocomposites, including onset temperature, mass loss of Steps 1 and 2 and residue in nitrogen and after switching to air.

Samples	Onset (°C)	Step 1 (wt.%)	Step 2 (wt.%)	Residue N_2_ (wt.%)	Residue Air (wt.%)
B60/40	337.46	28.57	70.92	0.50	0.50
B60/40_30PRE	299.49	24.43	69.65	4.67	2.69
B60/40_30POST	309.48	25.67	69.38	3.65	1.45
B60/40_30POST_AO	316.18	27.38	69.77	1.64	1.00
B30/70	336.24	32.30	68.17	0	0
B30/70_30PRE	305.75	30.24	68.49	0.51	0.50
B30/70_30POST	322.69	32.28	65.31	1.51	0.99
B30/70_30POST_AO	311.06	33.74	60.93	4.08	1.75

## Data Availability

The original contributions presented in this study are included in the article. Further inquiries can be directed to the corresponding author.
